# Global benchmarks for minimally invasive right hemicolectomy for cancer

**DOI:** 10.1093/bjs/znaf259

**Published:** 2025-12-16

**Authors:** Fariba Abbassi, Michaela Ramser, Matthias Pfister, Roxane D Staiger, Sun J Kim, Ji W Park, Bart C T van de Laar, Marcos Gonzalez, Vittoria Perano, Georgette Camilleri, David Merino, Justin Dourado, Anjelli Wignakumar, Kohei Shigeta, Tomás Mansur Duarte de Miranda Marques, Daniel Leonard, Kai-Yin Lee, Avanish Saklani, Kilian G M Brown, Fabio Butti, Ivana Raguz, Carlo Alberto Schena, Daichi Kitaguchi, Desmond C Winter, Masaaki Ito, Nicola de’Angelis, Dieter Hahnloser, Jennifer Vu, Ashwin Desouza, Bei-En Siew, Ker-Kan Tan, Alex Kartheuser, Samuel Aguiar, Koji Okabayashi, Carl J Brown, Steven Wexner, Sebastiano Biondo, Danilo Miskovic, Antonino Spinelli, Carlos A Vaccaro, Esther C J Consten, Byung S Min, Milo A Puhan, Matthias Turina

**Affiliations:** Department of Surgery and Transplantation, University of Zurich, Zurich, Switzerland; Institute of Epidemiology, Biostatistics and Prevention, University of Zurich, Zurich, Switzerland; Department of Visceral Surgery and Transplantation, University Hospital Zurich, Zurich, Switzerland; Wyss Zurich Translational Centre, University of Zurich and ETH Zurich, Zurich, Switzerland; Department of General and Abdominal Surgery, Cantonal Hospital Lucerne, Lucerne, Switzerland; Department of Surgery, Yonsei University College of Medicine and Severance Hospital, Seoul, South Korea; Department of Surgery, Seoul National University College of Medicine, Jongno-gu, South Korea; Department of Surgery, Meander Medical Centre, Amersfoort, The Netherlands; Department of Surgery, University Medical Centre Groningen, Groningen, The Netherlands; Section of Colorectal Surgery, Hospital Italiano de Buenos Aires and Instituto de Medicina Traslacioal e Ingeniería Biomédica (IMTIB), Buenos Aires, Argentina; Department of Biomedical Sciences, Humanitas University, Milan, Italy; Department of Colorectal Surgery, St Mark’s Hospital, London, UK; Department of General and Digestive Surgery and IDIBELL, Bellvitge University Hospital and University of Barcelona, Barcelona, Spain; Ellen Leifer Shulman and Steven Shulman Digestive Disease Centre, Cleveland Clinic Florida, Weston, Florida, USA; Department of Surgery, Florida Atlantic University, Boca Raton, Florida, USA; Ellen Leifer Shulman and Steven Shulman Digestive Disease Centre, Cleveland Clinic Florida, Weston, Florida, USA; Department of Surgery, Keio University School of Medicine, Tokyo, Japan; Colorectal Cancer Reference Centre, A. C. Carmago Cancer Centre, Sao Paulo, Brazil; Colorectal Surgery Unit, University Hospital Saint-Luc, Brussels, Belgium; Department of Colorectal Surgery, National University Health System, Singapore; Department of Surgical Oncology, Tata Memorial Hospital and Homi Bhabha National Institute, Mumbai, India; Department of Colorectal Surgery, Royal Prince Alfred Hospital, Sydney Local Health District, Sydney, New South Wales, Australia; Department of Visceral Surgery, Lausanne University Hospital, Lausanne, Switzerland; Department of Visceral Surgery and Transplantation, University Hospital Zurich, Zurich, Switzerland; Department of Colorectal Surgery, Beaujon Hospital and University of Paris, Clichy, France; Department of Colorectal Surgery, National Cancer Centre-Hospital East, Chiba, Japan; Department of Surgery, St Vincent’s Hospital, Dublin, Ireland; Department of Colorectal Surgery, National Cancer Centre-Hospital East, Chiba, Japan; Department of Colorectal Surgery, Beaujon Hospital and University of Paris, Clichy, France; Department of Visceral Surgery, Lausanne University Hospital, Lausanne, Switzerland; Department of Colorectal Surgery, Royal Prince Alfred Hospital, Sydney Local Health District, Sydney, New South Wales, Australia; Department of Surgical Oncology, Tata Memorial Hospital and Homi Bhabha National Institute, Mumbai, India; Department of Surgery, National University of Singapore Yong Loo Lin School of Medicine, Singapore; Department of Colorectal Surgery, National University Health System, Singapore; Department of Surgery, National University of Singapore Yong Loo Lin School of Medicine, Singapore; Colorectal Surgery Unit, University Hospital Saint-Luc, Brussels, Belgium; Colorectal Cancer Reference Centre, A. C. Carmago Cancer Centre, Sao Paulo, Brazil; Department of Surgery, Keio University School of Medicine, Tokyo, Japan; Section of Colorectal Surgery, University of British Columbia and St Paul’s Hospital, Vancouver, British Columbia, Canada; Ellen Leifer Shulman and Steven Shulman Digestive Disease Centre, Cleveland Clinic Florida, Weston, Florida, USA; Department of General and Digestive Surgery and IDIBELL, Bellvitge University Hospital and University of Barcelona, Barcelona, Spain; Department of Colorectal Surgery, St Mark’s Hospital, London, UK; Department of Biomedical Sciences, Humanitas University, Milan, Italy; Division of Colon and Rectal Surgery, Department of Surgery, IRCCS Humanitas Research Hospital, Milan, Italy; Section of Colorectal Surgery, Hospital Italiano de Buenos Aires and Instituto de Medicina Traslacioal e Ingeniería Biomédica (IMTIB), Buenos Aires, Argentina; Department of Surgery, Meander Medical Centre, Amersfoort, The Netherlands; Department of Surgery, University Medical Centre Groningen, Groningen, The Netherlands; Department of Surgery, Yonsei University College of Medicine and Severance Hospital, Seoul, South Korea; Institute of Epidemiology, Biostatistics and Prevention, University of Zurich, Zurich, Switzerland; Department of Visceral Surgery and Transplantation, University Hospital Zurich, Zurich, Switzerland

## Introduction

Colorectal cancer is the third most common cancer worldwide, representing approximately 10% of all newly diagnosed cancers, and is the second leading cause of cancer-related mortality^1^. Approximately 20% of colorectal cancers are located on the right side of the colon^2^ and can be treated by right hemicolectomy using oncological principles.

The principles of ‘oncological right hemicolectomy’ have been an area of recent focus. A more thorough understanding of the importance of meticulous dissection to achieve complete mesocolic excision (CME) and central (D3) lymphadenectomy may improve outcomes in patients with node-positive disease^3–5^. In addition, minimally invasive techniques such as laparoscopy and robot-assisted surgery have become the standard of care^6–8^.

In many countries, surgery for colonic cancer patients is not centralized or restricted to high-volume units. This contrasts with rectal cancer, where centralization and specialization have long been believed to be key factors in outcomes and quality of care^9–11^. Colonic cancer surgery is often performed by general surgeons in low-volume centres^12,13^. This is notable, given the established correlation between centre volume and better outcomes in colorectal surgery^13,14^. Consequently, continuous monitoring of surgical outcome quality is essential.

Various efforts have been made to evaluate and enhance the quality of colon cancer surgery. Previous classification systems primarily focused on measuring surgical results and outcomes through the evaluation of the surgical specimen. One of the earliest tools to validate the performance of adequate CME surgery was a pathological grading system introduced to assess the quality of colonic resections^15^. Similar to the assessment of total mesorectal excision (TME), this system rated the integrity of the surgical planes and the appropriateness of dissection levels. A subsequent classification, published by Benz *et al*.^16^, assessed the degree of radicality of resection by addressing missing mesocolic tissue and the surgical plane. However, both systems have important limitations, as neither of them accounts for patient factors, tumour stage, or surgical details. Therefore, a comprehensive quality assurance tool for right hemicolectomy is needed.

Benchmarking is a quality improvement process that involves identification of best practice and facilitation of performance and outcome comparisons against the highest achievable standards, while considering a wide range of influencing factors and parameters^17^. Initially a well-established tool for quality assessment in business and manufacturing, benchmarking is now increasingly adopted in surgical outcome research^18^. Several surgical procedures^19–25^ have been benchmarked according to a standardized approach that was validated through Delphi consensus^18,26^.

Benchmark cut-offs for outcome parameters are determined using ideal patients, that is low-risk patients who have undergone surgery at high-volume centres. Therefore, they specify the best achievable outcome for a specific procedure. These benchmarks serve as reference values and thus enable surgical outcome comparison. Centres and surgeons can compare their own performance to detect quality gaps to identify areas for improvement. Establishing benchmarks should lead to improved surgical quality and thus improved patient outcomes^26^.

Although benchmark cut-offs have been established for several surgical procedures, they are to date not available for right hemicolectomy. The aim of this study was to establish benchmark cut-offs for frequently used outcome parameters for elective minimally invasive right hemicolectomy for adenocarcinoma of the ascending colon.

## Methods

### Study design

To establish international valid benchmark cut-offs for right hemicolectomies in right-sided colon cancer, the well-established ten-step approach was utilized^18^. Carefully selected centres that met specific criteria were included^26^. Centres had to perform at least 250 colorectal resections annually or 100 per surgeon, maintain prospective data collection for oncological colorectal resections, conduct multidisciplinary tumour board discussions focused on colorectal tumours, and be actively involved in research in this field.

Colorectal specialist centres in Europe, North America, South America, Asia, and Australia were contacted based on these criteria by the investigators and invited to participate. The centres submitted pseudoanonymized data, free of patient identifiers, for all consecutive elective minimally invasive (laparoscopic or robotic) right hemicolectomies performed between July 2017 and June 2022 for adenocarcinoma of the coecum or ascending colon.

### Study cohort

Inclusion criteria for patients were an age of ≥18 years, confirmed adenocarcinoma in the right hemicolon, and a follow-up interval of at least 6 months. Exclusion criteria were pathologies other than adenocarcinoma, tumour location in the transverse colon, synchronous colorectal cancer at another site requiring additional resections other than a right hemicolectomy, and open surgery.

### Benchmarking

Patients were divided into two risk groups based on predefined parameters (*Table 1*). These were derived from established risk factors for postoperative medical and surgical complications following colorectal resection^27–31^. Patients at low risk of complications were classified as ‘ideal’, whereas those at high risk of complications were classified as ‘non-ideal’.

**Table 1:** Inclusion and exclusion criteria for ideal patients

Inclusion criteria	Exclusion criteria
Adult patients (≥18 years) Resectable adenocarcinoma of the right colon Elective surgery Tumour stage cT1–T3 No distant metastases (according to preoperative imaging, discovered intraoperatively, or according to the final histopathology) BMI ≥20 to <35 kg/m^2^	**Surgical reasons** Emergency procedures (performed within 24 h from emergency presentation) Preoperative bowel obstruction **Medical reasons** ASA grade ≥III Cardiac disease: Congestive heart failure onset or exacerbation in 30 days before surgery Myocardial infarction within 6 months before surgery History of percutaneous coronary intervention or cardiac surgery Atrial fibrillation Chronic renal failure, MDRD stage ≥3: GFR <60 ml/min/1.73 m^2^ or serum creatinine >1.8 mg/dl or 160 mmol/l Chronic obstructive pulmonary disease with FEV1 <80% Daily smoking within the last year before surgery Diabetes mellitus with ≥2 oral antidiabetic drugs or insulin Hypoalbuminaemia: preoperative albumin level <3.0 g/dl Use of anticoagulants (vitamin K antagonist, NOACs, clopidogrel) **Oncological reasons** Tumour stage cT4

MDRD, Modification of Diet in Renal Disease; GFR, glomerular filtration rate; FEV1, forced expiratory volume in 1 s; NOACs, non-vitamin K antagonist oral anticoagulants.

A total of 19 clinically relevant and widely used outcome measures were collected, including perioperative parameters, oncological parameters, procedure-specific complications, morbidity, and mortality (*Table S1*). Morbidity and mortality were assessed at hospital discharge, as well as at 3 and 6 months after surgery. Each complication was graded using the Clavien–Dindo classification (CDC)^32,33^, while a patient’s overall morbidity was summarized using the Comprehensive Complication Index^®^ (CCI^®^)^34,35^. The selection of outcome parameters was based on their clinical relevance, consistent use in previous benchmark studies, and alignment with international guidelines, as well as the recent consensus recommendations on outcome reporting in surgery^36–38^.

The benchmark cut-offs were derived from the ideal patient cohort of each individual centre. To prevent centres with a small number of ideal patients from disproportionately influencing the results of this study, a minimum of ten ideal patients per centre was required. Centres contributing fewer than ten ideal patients were included in the analysis only for the non-ideal cohort.

### Statistical analysis

Data were collected in Excel (Microsoft, Remond, WA, USA). All analyses were performed using R Statistical Software version 4.3.2 (R Core Team, Vienna, Austria)^39^. Discrete variables are presented as *n* (%) and continuous variables are presented as median (interquartile range (i.q.r.)).

Benchmark cut-off calculations were performed according to the previously published definition and in accordance with the Delphi agreement of experts^18,26^. In short, benchmarks were set at the 75th percentile of all centres’ median values for negative outcomes and at the 25th percentile of all centres’ median values for positive outcomes.

### Ethical approval

Ethical approval was obtained from the Canton Zurich, Switzerland (BASEC 2022-01200), as well as by each participating centre according to local regulations.

## Results

### Baseline data

A total of 21 centres from six continents submitted data on eligible patients.

Two centres were excluded from the analysis due to protocol violations or incomplete data sets with missing parameters needed to define ideal and non-ideal patients. Another two centres did not meet the minimum requirement of ten ideal patients, making only their non-ideal patients eligible for inclusion.

As a result, 17 centres from five continents (6 centres from Europe, 2 centres from North America, 2 centres from South America, 6 centres from Asia, and 1 centre from Australia) were included in the benchmark analysis for ideal patients, and 19 centres contributed to the non-ideal cohort.

**Fig. 1:**
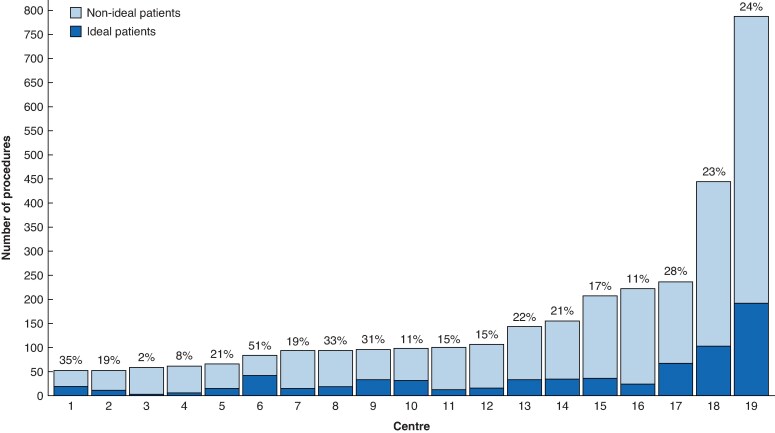
Distribution of minimally invasive right hemicolectomies across centres Among 19 reference centres, a total of 3154 patients were analysed, of whom 686 (21.8%) were ideal patients and 2468 (78.2%) were non-ideal patients. The percentage of ideal patients for each centre is shown; this varied widely among centres, ranging from 1.7% to 51.2%.

Overall, 3154 patients were analysed, of whom 686 (21.8%) were ideal patients and 2468 (78.2%) were non-ideal patients. The proportion of ideal patients varied widely among centres, ranging from 1.7% to 51.2% (*Fig. 1*). Baseline characteristics for the overall cohort, as well as for ideal and non-ideal patients, are presented in *Table 2*. The ideal patient group was predominantly female (372 of 686 (54.2%)), had a median age of 64 (i.q.r. 56–71) years, and had a median BMI of 24.5 (i.q.r. 22.3–27.0) kg/m^2^.

**Table 2:** Baseline characteristics

	Overall (*n* = 3154)	Ideal patients (*n* = 686)	Non-ideal patients (*n* = 2468)
Age (years), median (i.q.r.)	71 (61–78)	64 (56–71)	73 (64–80)
**Sex**			
Male	1557 (49.4)	314 (45.8)	1243 (50.4)
Female	1597 (50.6)	372 (54.2)	1225 (49.6)
BMI (kg/m^2^), median (i.q.r.)	24.5 (22.0–27.5)	24.5 (22.3–27.0)	24.5 (22.0–27.7)
Congestive heart failure	61 (1.9)	0 (0.0)	61 (2.5)
Myocardial infarction	56 (1.8)	0 (0.0)	56 (2.3)
Cardiac intervention/surgery	276 (8.8)	0 (0.0)	276 (11.2)
Atrial fibrillation	234 (7.4)	0 (0.0)	234 (9.5)
Chronic renal failure	216 (6.8)	0 (0.0)	216 (8.8)
COPD	143 (4.5)	0 (0.0)	143 (5.8)
Diabetes	535 (17.0)	0 (0.0)	535 (21.7)
Immunosuppression	61 (1.9)	0 (0.0)	61 (2.5)
Smoking	413 (13.1)	0 (0.0)	413 (16.7)
Anticoagulation	309 (9.8)	0 (0.0)	309 (12.5)
**ASA grade**			
I	274 (8.7)	152 (22.2)	122 (4.9)
II	1713 (54.3)	534 (77.8)	1179 (47.8)
III	1096 (34.7)	0 (0.0)	1096 (44.4)
IV	71 (2.3)	0 (0.0)	71 (2.9)
History of major abdominal surgery*	714 (22.6)	0 (0.0)	714 (28.9)
Diagnosis to surgery (days), median (i.q.r.)	27 (14–44)	25 (13–42)	27 (14–44)
Preoperative chemotherapy	80 (2.5)	0 (0.0)	80 (3.2)
Preoperative radiotherapy	2 (<0.1)	0 (0.0)	2 (<0.1)
Liver-first approach	13 (0.4)	0 (0.0)	13 (0.5)
Mechanical bowel preparation	1902 (60.7)	411 (60.1)	1491 (60.9)
Perioperative intravenous antibiotics	2668 (84.9)	584 (85.1)	2084 (84.8)
Emergency surgery	64 (2.0)	0 (0.0)	64 (2.6)
**Surgical approach**			
Laparoscopic	2933 (93.1)	645 (94.0)	2288 (92.8)
Robotic	219 (6.9)	41 (6.0)	178 (7.2)
**Surgical radicality**			
CME	1818 (57.7)	423 (61.8)	1395 (56.6)
D2 LA	1174 (37.3)	232 (33.9)	942 (38.2)
D3 LA	159 (5.0)	30 (4.4)	129 (5.2)
**Anastomosis**			
Extracorporeal	2446 (79.1)	531 (78.7)	1915 (79.2)
Intracorporeal	646 (20.9)	144 (21.3)	502 (20.8)
**Anastomotic technique**			
Stapled	2729 (88.9)	630 (93.5)	2099 (87.6)
Handsewn	332 (10.8)	44 (6.5)	288 (12.0)
Ostomy	10 (0.3)	0 (0.0)	10 (0.4)
**Tumour stage**			
pTis	17 (0.5)	0 (0.0)	17 (0.7)
pT1	369 (11.7)	117 (17.1)	252 (10.2)
pT2	480 (15.2)	142 (20.7)	338 (13.7)
pT3	1779 (56.4)	427 (62.2)	1352 (54.8)
pT4	495 (15.7)	0 (0.0)	495 (20.1)
No residual tumour	14 (0.4)	0 (0.0)	14 (0.6)
**Nodes stage**			
pN0	2008 (63.7)	464 (67.6)	1544 (62.6)
pN1	785 (24.9)	156 (22.7)	629 (25.5)
pN2	359 (11.4)	66 (9.6)	293 (11.9)
pN3	1 (<0.1)	0 (0.0)	1 (<0.1)
**Metastases stage**			
c/pM0	2911 (92.3)	686 (100.0)	2225 (90.2)
c/pM1	243 (7.7)	0 (0.0)	243 (9.8)

Values are *n* (%) unless otherwise indicated. *Defined as others than diagnostic laparoscopy, laparoscopic appendectomy, laparoscopic cholecystectomy, laparoscopic adnexectomy. COPD, chronic obstructive pulmonary disease; CME, complete mesocolic excision; LA, lymphadenectomy; Tis, carcinoma *in situ*.

### Validation of the ideal criteria

To validate the relevance of the selected parameters for distinguishing between ideal and non-ideal patients, postoperative outcomes were compared between the two cohorts (*Table 3*). Key parameters, such as length of hospital stay, R0 resection rate, number of resected lymph nodes, and complication rates, all showed statistically significant differences.

**Table 3:** Cohort validation

Outcome parameter	Ideal patients (*n* = 686)	Non-ideal patients (*n* = 2468)	P*
Duration of surgery (min), median (i.q.r.)	170 (130–210)	172 (131–218)	0.304
Conversion to open	20 (2.9)	134 (5.4)	0.007†
ICU stay ≥3 days	6 (1.0)	74 (3.5)	0.002†
R0 resection	685 (99.9)	2385 (97.0)	<0.001†
Number of lymph nodes harvested, median (i.q.r.)	27 (20–37)	24 (18–32)	<0.001†
≥12 lymph nodes harvested	673 (98.4)	2365 (96.5)	0.013†
Anastomotic leak	14 (2.0)	62 (2.5)	0.475
Pancreatic fistula	1 (0.1)	7 (0.3)	>0.999
Duodenal leak	1 (0.1)	1 (<0.1)	0.388
Bleeding requiring transfusion	19 (2.8)	135 (5.5)	0.004†
Bleeding requiring surgery	1 (0.1)	11 (0.4)	0.482
Ureteric injury	0 (0.0)	1 (<0.1)	>0.999
Deep SSI	25 (3.6)	85 (3.4)	0.803
Length of hospital stay (days), median (i.q.r.)	5 (4–7)	6 (4–8)	<0.001†
Hospital readmission	29 (4.2)	197 (8.0)	<0.001†
**At discharge**			
Any complication	138 (20.1)	745 (30.2)	<0.001†
Major complication (CDC grade ≥IIIa)	22 (3.2)	132 (5.4)	0.021†
CCI^®^, median (i.q.r.)	0 (0–0)	0 (0–8.7)	<0.001†
CCI^®^, mean(s.d.)	4.2 (9.9)	6.9 (13.6)	<0.001†
Mortality	1 (0.1)	15 (0.6)	0.220
**At 3 months**			
Any complication	181 (26.5)	897 (36.4)	<0.001†
Major complication (CDC grade ≥IIIa)	31 (4.5)	181 (7.4)	0.009†
CCI^®^, median (i.q.r.)	0 (0–8.7)	0 (0–20.9)	<0.001†
CCI^®^, mean(s.d.)	5.7 (12.1)	9.0 (16.3)	<0.001†
Mortality	2 (0.3)	18 (0.7)	0.280
**At 6 months**			
Any complication	187 (27.3)	921 (37.6)	<0.001†
Major complication (CDC grade ≥IIIa)	34 (5.0)	208 (8.5)	0.002†
CCI^®^, median (i.q.r.)	0 (0–8.7)	0 (0–20.9)	<0.001†
CCI^®^, mean(s.d.)	6.2 (13.7)	10.0 (18.7)	<0.001†
Mortality	3 (0.4)	26 (1.1)	0.127

Values are *n* (%) unless otherwise indicated. *Wilcoxon’s rank-sum test; Pearson’s chi-squared test; Fisher’s exact test. †Statistically significant. SSI, surgical-site infection; CDC, Clavien–Dindo classification; CCI^®^, Comprehensive Complication Index^®^.

### Benchmark cut-offs

Data from 686 ideal patients were used to establish benchmark cut-offs, which are listed in *Table 4*. Important benchmark cut-offs were an R0 resection rate of 100.0%, ≥96.9% of patients with ≥12 lymph nodes harvested, ≥23 lymph nodes harvested, and an anastomotic leak rate of ≤3.0%. At discharge, important benchmark cut-offs were a major complication (CDC grade ≥IIIa) rate of ≤5.6%, a median CCI^®^ of 0, and a mortality of 0.0%.

**Table 4:** Benchmark cut-off values for minimally invasive right hemicolectomy

Outcome parameter	Benchmark cut-off values*
Duration of surgery (min)	≤210
Conversion to open	≤5.7
ICU stay ≥3 days	≤1.7
Length of hospital stay (days)	≤6
R0 resection	100.0
Number of lymph nodes harvested	≥23
≥12 lymph nodes harvested	≥96.9
Anastomotic leak	≤3.0
Pancreatic fistula	0.0
Duodenal leak	0.0
Bleeding requiring transfusion	≤4.5
Bleeding requiring surgery	0.0
Ureteric injury	0.0
Deep SSI	≤5.6
Hospital readmission	≤6.3
**At discharge**	
Any complication	≤28.6
Major complication (CDC grade ≥IIIa)	≤5.6
Median CCI^®^	0
Mean CCI^®^	≤0
Mortality	0.0
**At 3 months**	
Any complication	≤37.9
Major complication (CDC grade ≥IIIa)	≤8.3
Median CCI^®^	0
Mean CCI^®^	≤2
Mortality	0.0
**At 6 months**	
Any complication	≤37.9
Major complication (CDC grade ≥IIIa)	≤8.3
Median CCI^®^	0
Mean CCI^®^	≤3
Mortality	0.0

Values are % unless otherwise indicated. *Benchmarks were set at the 75th percentile of all centres’ median values for negative outcomes and at the 25th percentile of all centres’ median values for positive outcomes. SSI, surgical-site infection; CDC, Clavien–Dindo classification; CCI^®^, Comprehensive Complication Index^®^.

### Centre volume and outcomes

To assess the impact of centre volume on outcomes and quality, the participating reference centres were categorized into two groups: those performing ≥500 colorectal resections annually and those performing 250 to <500 colorectal resections annually. Data were analysed separately for the ideal and non-ideal subpopulations. Although all of the centres were high-volume specialized colorectal units, significant differences were observed regarding the number of lymph nodes harvested, complication rates and CCI^®^, with centres performing ≥500 resections demonstrating superior results (*Table 5*).

**Table 5:** Impact of centre volume on surgical outcomes in ideal and non-ideal patients

	Ideal patients			Non-ideal patients		
	250 to <500 resections/year (*n* = 219)	≥500 resections/year (*n* = 467)	P*	250 to <500 resections/year (*n* = 937)	≥500 resections/year (*n* = 1531)	P*
Duration of surgery (min), median (i.q.r.)	170 (108–225)	170 (135–204)	0.119	180 (119–220)	170 (137–216)	0.011†
Conversion to open	8 (3.7)	12 (2.6)	0.432	53 (5.7)	81 (5.3)	0.703
ICU stay ≥3 days	2 (0.9)	4 (1.1)	>0.999	40 (4.3)	34 (2.9)	0.076
R0 resection	218 (99.5)	467 (100.0)	0.319	907 (96.9)	1478 (97.0)	0.840
Number of lymph nodes harvested, median (i.q.r.)	25 (19–34)	29 (21–38)	0.003†	21 (16–28)	26 (19–34)	<0.001†
≥12 lymph nodes harvested	212 (96.8)	461 (99.1)	0.044†	884 (94.3)	1 481 (97.9)	<0.001†
Anastomotic leak	7 (3.2)	7 (1.5)	0.155	28 (3.0)	34 (2.2)	0.239
Pancreatic fistula	0 (0.0)	1 (0.2)	>0.999	1 (0.1)	6 (0.4)	0.264
Duodenal leak	0 (0.0)	1 (0.2)	>0.999	1 (0.1)	0 (0.0)	0.380
Bleeding requiring transfusion	7 (3.2)	12 (2.6)	0.641	55 (5.9)	80 (5.2)	0.499
Bleeding requiring surgery	1 (0.5)	0 (0.0)	0.319	5 (0.5)	6 (0.4)	0.757
Ureteric injury	0 (0.0)	0 (0.0)	–	0 (0.0)	1 (<0.1)	>0.999
Deep SSI	8 (3.7)	17 (3.6)	0.993	28 (3.0)	57 (3.7)	0.328
Length of hospital stay (days), median (i.q.r.)	4 (3–7)	5 (5–7)	<0.001†	5 (4–8)	6 (5–8)	<0.001†
Hospital readmission	11 (5.0)	18 (3.9)	0.478	76 (8.1)	121 (7.9)	0.840
**At discharge**						
Any complication	59 (26.9)	79 (16.9)	0.002†	371 (39.6)	374 (24.5)	<0.001†
Major complication (CDC grade ≥IIIa)	14 (6.4)	8 (1.7)	0.002†	65 (6.9)	67 (4.4)	0.006†
CCI^®^, median (i.q.r.)	0 (0–9)	0 (0–0)	0.001†	0 (0–21)	0 (0–0)	<0.001†
CCI^®^, mean(s.d.)	6.0 (11.7)	3.3 (8.8)	0.001†	9.4 (16.1)	5.4 (11.6)	<0.001†
Mortality	0 (0.0)	1 (0.2)	>0.999	9 (1.0)	6 (0.4)	0.078
**At 3 months**						
Any complication	72 (32.9)	109 (23.4)	0.009†	423 (45.2)	474 (31.1)	<0.001†
Major complication (CDC grade ≥IIIa)	17 (7.8)	14 (3.0)	0.006†	86 (9.2)	95 (6.3)	0.006†
CCI^®^, median (i.q.r.)	0 (0–9)	0 (0–0)	0.002†	0 (0–21)	0 (0–9)	<0.001†
CCI^®^, mean(s.d.)	7.9 (14.3)	4.7 (10.8)	0.002†	11.8 (19.2)	7.2 (13.9)	<0.001†
Mortality	1 (0.5)	1 (0.2)	>0.999	12 (1.3)	6 (0.4)	0.012†
**At 6 months**						
Any complication	76 (34.7)	111 (23.9)	0.003†	433 (46.8)	488 (32.0)	<0.001†
Major complication (CDC grade ≥IIIa)	18 (8.2)	16 (3.5)	0.008†	95 (10.4)	113 (7.4)	0.012†
CCI^®^, median (i.q.r.)	0 (0–17)	0 (0–0)	<0.001†	0 (0–21)	0 (0–10)	<0.001†
CCI^®^, mean(s.d.)	8.7 (15.7)	5.1 (12.5)	<0.001†	13.1 (21.8)	8.1 (16.2)	<0.001†
Mortality	1 (0.5)	2 (0.4)	>0.999	14 (1.6)	12 (0.8)	0.079

Values are *n* (%) unless otherwise indicated. *Wilcoxon’s rank-sum test; Pearson’s chi-squared test; Fisher’s exact test. †Statistically significant. i.q.r., interquartile range; SSI, surgical-site infection; CDC, Clavien–Dindo classification; CCI^®^, Comprehensive Complication Index^®^.

## Discussion

This international, multicentre study establishes benchmarks for oncological minimally invasive right hemicolectomy using a well-recognized benchmark methodology^18,26^. It identifies the best achievable surgical outcomes for patients with adenocarcinoma of the right hemicolon treated at high-volume colorectal reference centres worldwide. These benchmark cut-offs encompass perioperative parameters, oncological quality indicators, procedure-specific complications, overall morbidity, and mortality. They serve as a reference for comparing data from individual surgeons, centres, and registries that should aid the improvement of healthcare quality.

The excellent results for ideal patients confirm the high quality achieved in specialized centres. Oncological quality indicators show an R0 resection rate of almost 100% for ideal patients. In addition, the median number of resected lymph nodes was 25 and therefore well above the required minimum. Further indicators of high quality are the low anastomotic leak rate of only 2%, as well as the low conversion rate and the short length of hospital stay.

The high rate of reported complications is remarkable, with a complication rate of 20.1% in ideal patients and a comparatively low rate of severe complications (CDC grade ≥III) of 3.2%. These data suggest that the prospective databases of the participating centres were maintained to a high standard, as many CDC grade I and II complications were registered.

The most recent trial reporting outcomes after elective oncological minimally invasive right hemicolectomies is the observational MIRCAST study^40^; the anastomotic leak rates ranged from 0.5% to 2.1% across the different surgical approaches and anastomotic techniques and were close to that for the benchmark cohort in the present study (median anastomotic leak rate of 2.0%; cut-off value of ≤3.0%).

Suspected procedure-specific complications such as pancreatic fistulas, duodenal leak, or major bleeding, all of which were widely discussed upon introduction of CME surgery, were rare in both ideal and non-ideal patients in the present analysis.

The differences in intraoperative bleeding between CME and D2 lymphadenectomy observed in the RELARC trial may be explained by the extent of lymphadenectomy performed around the superior mesenteric artery, an area not typically included in standard CME, which usually extends only to the superior mesenteric vein^7^. This example underscores the issue of inconsistent definitions and interpretation of D2/D3 lymphadenectomy in the literature and clinical practice. For this reason, in the present study, the authors deliberately refrained from including CME, D2 lymphadenectomy, or D3 lymphadenectomy as an outcome parameter.

The benchmark cut-off for conversion to open surgery was 5.7%. Although the reasons for conversion were not explored, the rate of laparoscopically performed right hemicolectomies might be a possible explanation (94.0% of procedures were performed laparoscopically in ideal patients). It is well known that conversion rates for robot-assisted procedures are lower. Spinoglio *et al*.^41^ published conversion rates of 0% for robot-assisted right hemicolectomies compared with 6.9% for laparoscopic procedures. In the present cohort, only 6.0% of ideal patients and 7.2% of non-ideal patients were operated robotically. Therefore, most centres were still using the laparoscopic approach or might still have been in the learning curve phase for robotic surgery.

Although the impact of specialization and centralization is well established, and all included centres met the criteria for high-volume colorectal reference centres, there was a difference in outcomes among these top-tier hospitals, with better results associated with very high-volume centres. This probably reflects the additional effect of standardization and cumulative experience. There is no established threshold or ceiling effect, rendering the definition of high-volume colorectal reference centres inherently arbitrary by design^42^.

The limitation of the present study is the focus on minimally invasive procedures. Centres were asked to submit data on minimally invasive procedures only, assuming that the rate of procedures performed using an open technique does not change over time once a high-volume minimally invasive colonic surgery programme is established and only patient or tumour factors will lead to the performance of open surgery^43^.

A further consideration is the applicability of these benchmarks in different healthcare settings. To ensure realistic and reproducible results, benchmark cut-offs are not based on single-centre outliers, but are conservatively set at the 75th percentile of all centres’ median values for negative outcomes and at the 25th percentile of all centres’ median values for positive outcomes. In many low-income countries, oncological right hemicolectomies are still predominantly performed using an open approach, which was not the focus of this analysis. However, once the necessary infrastructure for minimally invasive surgery is established, the same benchmark values should apply irrespective of geographical or economic context, providing a valuable tool for performance evaluation and quality improvement even in resource-limited environments.

This study establishes benchmark values for outcome parameters of minimally invasive right hemicolectomy that can be used as a performance reference for the evaluation of surgical care.

## Supplementary Material

znaf259_Supplementary_Data

## Data Availability

The data supporting this paper are available from the corresponding author upon reasonable request.
