# Supplementary material for: Global benchmarks for minimally invasive right hemicolectomy for cancer
Source: Br J Surg. 2025 Dec 16;112(12):znaf259. doi: 10.1093/bjs/znaf259 (PMC12706472; doi:10.1093/bjs/znaf259)
Supplement: znaf259_Supplementary_Data [file znaf259_supplementary_data.docx]

**Global Benchmarks for
Minimally Invasive Right Hemicolectomy for Cancer**

Fariba Abbassi, MD^1,2^; Michaela Ramser, MD^3^; Matthias Pfister, MD^4^, Roxane D Staiger, MD, PhD^5^; Sun Jung Kim, MD^6^; Ji Won Park, MD, PhD^7^; Bart CT van de Laar, MD^8, 9^; Marcos Gonzalez, MD, PhD^10^; Vittoria Perano, MD^11^; Georgette Camilleri, MD^12^; David Merino, MD^13^; Justin Dourado, MD^14,15^; Anjelli Wignakumar, MD^14^; Navindi De Silva^16^; Kohei Shigeta, MD, PhD^17^; Tomás Mansur Duarte de Miranda Marques, MD^18^; Daniel Leonard, MD, PhD^19^; Kai-Yin Lee, MD^20^; Avanish Saklani, MD^21^; Kilian GM Brown, MBBS^22^; Fabio Butti, MD^23^; Ivana Raguz, MD^3^; Carlo Alberto Schena, MD^24^; Daichi Kitaguchi, MD, PhD^25^; Desmond C Winter, MD, PhD^26^; Masaaki Ito, MD, PhD^25^; Nicola de’Angelis, MD, PhD^24^; Dieter Hahnloser, MD^23^; Jennifer Vu, MD^22^; Ashwin deSouza, Mch^21^; Bei-En Siew^27^; Ker-Kan Tan, MD, PhD^20,27^; Alex Kartheuser, MD, PhD^19^; Samuel Aguiar JR, MD^18^; Koji Okabayashi, MD^17^; Carl Brown, MD, PhD^16^; Steven D Wexner, MD, PhD^14^; Sebastiano Biondo, MD, PhD^13^; Danilo Miskovic, MD, PhD^12^; Antonino Spinelli, MD, PhD^11,28^; Carlos A Vaccaro, MD, PhD^10^; Esther CJ Consten, MD, PhD^8,9^; Byung Soh Min, MD, PhD^6^; Milo A Puhan, MD, PhD^2^; Matthias Turina, MD, PhD^3^
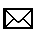


*^1^Department of Surgery and Transplantation, University of Zurich, Zurich (Switzerland)
^2^Epidemiology, Biostatistics and Prevention Institute, University of Zurich, Zurich (Switzerland)
^3^Department of Abdominal Surgery and Transplantation, University Hospital Zurich, Zurich (Switzerland)
^4^Wyss Zurich Translational Centre, ETH Zurich and University of Zurich, Zurich (Switzerland)
^5^Department of General and Visceral Surgery, Lucerne Cantonal Hospital, Lucerne (Switzerland)
^6^Department of Surgery, Colorectal Cancer Clinic, Severance Hospital, Yonsei University College of Medicine, Seoul (Korea)
^7^Department of Surgery, Seoul National University College of Medicine, Seoul (Korea)
^8^Department of Surgery, Meander Medical Centre, Amersfoort (The Netherlands)
^9^Department of Surgery, University medical Centre Groningen, Groningen (The Netherlands)
^10^Section of Colorectal Surgery, Hospital Italiano de Buenos Aires and Instituto de Medicina Traslacional e Ingeniería Biomédica (IMTIB), Buenos Aires (Argentina)
^11^* *Department of Biomedical Sciences, Humanitas University, Pieve Emanuele, Milan (Italy)
^12^Department of Colorectal Surgery, St Mark's Hospital, Northwick Park, Harrow (United Kingdom)
^13^Bellvitge University Hospital, Department of General and Digestive Surgery, and IDIBELL, University of Barcelona, Barcelona (Spain)
^14^Ellen Leifer Shulman and Steven Shulman Digestive Disease Centre, Cleveland Clinic Florida, Weston (USA)
^15^Department of Surgery, Florida Atlantic University, Boca Raton (USA)*

*^16^Department of Surgery, University of British Columbia, St Paul's Hospital, Vancouver (Canada)
^17^Department of Surgery, Keio University School of Medicine, Tokyo (Japan)
^18^Colorectal Cancer Reference Centre, A.C. Camargo Cancer Centre, São Paulo City (Brazil)
^19^Colorectal Surgery Unit, University Hospitals Saint-Luc, Brussels (Belgium) ^20^Department of Colorectal Surgery, University Surgical Cluster, National University Health System, Singapore (Singapore)
^21^Department of Surgical Oncology, Tata Memorial Hospital and Homi Bhabha National Institute, Mumbai (India)
^22^Department of Colorectal Surgery, Royal Prince Alfred Hospital, Sydney (Australia)
^23^Department of Visceral Surgery, University Hospital Lausanne, Lausanne (Switzerland)
^24^Department of Colorectal Surgery, Beaujon Hospital and University of Paris, Clichy (France)
^25^Department of Colorectal Surgery, National Cancer Centre Hospital East, Chiba (Japan)
^26^St Vincent's University Hospital, Dublin (Ireland)
^27^Department of Surgery, Yong Loo Lin School of Medicine, National University of Singapore, (Singapore)
^28^IRCCS Humanitas Research Hospital, Rozzano, Milan (Italy)*


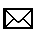
 Corresponding author: Matthias Turina, MD, PhD

*Department of Visceral Surgery and Transplantation*

*Rämistrasse 100*

*8044 Zurich, Switzerland*

*Email:*[*matthias.turina@usz.ch*](mailto:matthias.turina@usz.ch)

| **Supplementary Methods** |  |
| --- | --- |
| Table S1: Outcome Parameter for Minimally Invasive Right Hemicolectomy | *page 4* |

**Supplementary Methods**

Table S1. Outcome Parameter for Minimally Invasive Right Hemicolectomy

Perioperative parameters:

- Duration of surgery (minutes)
- Estimated blood loss (ml)
- Conversion to open surgery
- ICU stay (days)
- Length of hospital stay (days)

Procedure-specific complications:

- Anastomotic leak
- Postoperative bowel paralysis
- Bleeding requiring transfusion of RBC
- Surgical site infection
- Injury of the ureter
- Pancreatic fistula
- Duodenal leakage

Morbidity and Mortality:

- Clavien-Dindo grade of every complication at discharge, 3 months, and 6 months
- Readmission
- Re-operation
- CCI® at discharge, 3 months, and 6 months
- Postoperative mortality at discharge, 3 months, and 6 months

Oncologic quality:

- R0 resection
- Lymph node yield

ICU: intensive care unit, RBC: red blood cells; CCI: comprehensive complication index®
